# Electrophoretic Plasma Protein Reference Intervals for Backyard Chickens From Northern Colorado

**DOI:** 10.1111/vcp.70066

**Published:** 2025-10-27

**Authors:** Jeffrey Brandon, Heather Reider, Kristy L. Pabilonia, A Russell Moore

**Affiliations:** ^1^ Department of Microbiology, Immunology and Pathology Colorado State University Fort Collins Colorado USA; ^2^ Veterinary Diagnostic Laboratories Colorado State University Fort Collins Colorado USA

**Keywords:** albumin, electrophoresis, *Gallus gallus domesticus*, globulin, reference values

## Abstract

**Background:**

The number of backyard chicken ( *Gallus gallus domesticus* ) flocks in the US in urban and suburban areas has continued to increase over the past three decades. These flocks are often raised as both production animals and as pets. Electrophoretic evaluation of plasma proteins has been held as the reference standard for protein evaluation. Reference intervals (RIs) for plasma protein electrophoresis (PPE) in chickens in northern Colorado are not readily available but could aid diagnostic evaluations.

**Objectives:**

We aimed to generate RIs for PPE in adult non‐molting backyard chicken flocks in northern Colorado.

**Methods:**

Plasma from 120 healthy adult chickens in 7 flocks located in northern Colorado was used. Electrophoretic evaluation was completed using a biuret total protein assay and agarose gel electrophoresis, with proteins fractionated into 6 parts. RIs for PPE fractions were generated, and partitioning by sex was performed according to ASVCP guidelines.

**Results:**

RIs were generated for all birds and after partitioning by sex. For all birds, agarose gel electrophoretic RIs were: A:G 0.24–1.20, albumin 1.33–2.91 g/dL, alpha‐1 globulin 0.13–0.47 g/dL, alpha‐2 globulin 0.33–0.91 g/dL, beta‐1 globulin 0.24–1.50 g/dL, beta‐2 globulin 0.33–1.79 g/dL, and gamma globulin 0.26–0.82 g/dL. A statistical difference, *p* < 0.05, was noted between hens and roosters for all electrophoretic fractions except total alpha globulin, beta‐2 globulin, and A:G, and partitioning was warranted based on the method recommended by Lahti; the data were partitioned by sex, and RIs were generated.

**Conclusions:**

RIs are available for PPE in backyard chickens in northern Colorado.

## 1 Introduction

The number of backyard chicken ( *Gallus gallus domesticus* ) flocks in the US in urban and suburban areas has continued to increase over the past three decades. These flocks are often raised as both production animals and as pets [1]. This shift has expanded backyard poultry diagnostic medicine to include more ante‐mortem diagnostic evaluations. We have previously reported reference intervals (RIs) for plasma biochemical measurands assessed on reference laboratory equipment in mixed‐breed backyard chicken flocks in northern Colorado [2, 3]. Those RIs did not include electrophoretic assessment of plasma proteins.

Protein electrophoresis has long been held as the reference standard for the assessment of protein fractions, including albumin in birds [4, 5, 6, 7]. Reference intervals are available for dye‐binding protein fractions in chickens and other avian species [3, 6, 8, 9, 10, 11]. While some data are available for protein electrophoretic fractions in chickens, these have not been developed using current statistical approaches and electrophoretic techniques [12, 13, 14, 15]. Data suggest that age, sex, laying status, and molt impact electrophoretic protein fractions and interpretation [12, 16, 17]. In many backyard flocks, egg‐laying status is not always known, but molt can be observed visually. Therefore, the objective of this study was to generate reference intervals (RIs) for plasma protein agarose gel electrophoresis (PPE) fractions in non‐molting adult backyard chickens with unknown egg‐laying status and housed in northern Colorado according to current ASVCP guidelines [18].

## 2 Methods

This study was approved by the internal animal care and use committee at Colorado State University.

### 2.1 Analytical Performance Characterization

All methods were shown to be operating within expected quality parameters as defined in the laboratory. For the biuret total protein (TP) assay, the laboratory's routine commercial quality control materials (QCM, Bio‐Rad Multiqual 1 and Multiqual 3; Bio‐Rad, Hercules, CA, USA) were used. Verification of analytic performance in chickens was completed using duplicate measurements of 15 chicken plasma samples across runs and the within‐subject standard deviation method [19]. The analytic performance of electrophoretic measurands is typically evaluated using pooled canine and feline serum as QCM in our lab; verification of analytic performance in chickens was completed using 5 repeat measurements of a single chicken plasma sample.

Current ASVCP guidelines recommend a minimum of 120 subjects to determine nonparametric reference intervals and 90% confidence intervals (CI) [18]. A sample size of 150 was targeted to account for outliers and analytical errors. Samples were successfully collected from 131 adult non‐molting chickens during the summer months, between May 23, 2023, and July 18, 2023, of which 11 did not meet all exclusion criteria (i.e., had hemolysis or lipemia) or had visually aberrant electrophoretic profiles. These were excluded. 120 samples were available for RI generation. Twenty‐eight different non‐commercial breeds were represented, including 96 hens (80%) and 24 roosters (20%). Breeds represented by two or more individuals are listed in Table 1. The chickens used in this study were older than 4 months of age. Seven privately owned backyard flocks in northern Colorado (< 200 miles from Fort Collins, CO, at an altitude of 5000 ft), all participating in Colorado's National Poultry Improvement Plan (NPIP), consented to participate in this study. The seven flocks ranged in size from 45 to 150 birds, with a mean of 88 birds per flock. Birds were kept in coops with either access to enclosed runs or were free‐range.

**TABLE 1:** Summary of breeds with greater than 2 individuals in the reference population.

Breed	Count
Black Copper Marans	37
Black Australorp	14
Rhode Island Red	10
Black Cochin	8
Backyard Mixed Breed	8
Ameraucana	7
White Crested Black Polish	6
Blue Ameraucana	5
Austra White	3
Ayam Cemani	2
Sumatra	2
Braham	2
Other breeds	16

From each flock, a subset of birds was randomly selected after being determined to be clinically healthy, free from signs of molting or visible adult ectoparasites, and without signs of specific diseases based on history, physical examination, and were negative for the NPIP disease surveillance assays for pullorum–typhoid disease and avian influenza virus. The physical examination included an examination of the mouth, head, body, and extremities. The physical exam findings, sex, breed, and volume of blood sampled were recorded for each bird at the time of sample collection. Flock location, overall health, flock size, and diet (primarily consisting of commercially available layer feeds with occasional table scraps, chicken scratch, and/or supplements) were also documented.

Venipuncture of the brachial vein was performed using a 3 mL syringe and a 22‐gauge needle. The birds were manually restrained during venipuncture to collect 1–2 mL of blood. Samples were immediately transferred to heparinized vacutainers post‐collection, gently mixed, and stored in an insulated cooler with ice packs. Blood samples were maintained between 2°C–8°C during transport to the Colorado State University Clinical Pathology Laboratory, with all samples submitted within four hours from the time of collection, where the samples were centrifuged at 1500*g* for 10 min.

Biuret total protein (TP) and agarose gel protein electrophoresis were performed on all samples. The TP assay was performed on a commercial biochemistry analyzer using commercially available reagents (Cobas c501; Roche Diagnostics) at the time of submission. An H‐index > 125 or L‐index > 100, as assessed by the Cobas c501, was used as an exclusion criterion. Samples were stored at −20°C and electrophoresis was performed in batches, avoiding multiple freeze–thaw cycles. Protein electrophoresis was performed using non‐reduced amido black stained agarose gel electrophoresis (Sebia Hydrasys with Hydragel Protein (E) with amido black kit, Sebia Inc., Norcross, GA, USA). All electrophoretic evaluations were completed within 3 months of sample collection. Protein fractions were demarcated based on published data [20].

Reference Intervals were calculated, and outliers were identified using the Dixon–Reed and Tukey's tests, with emphasis on retaining results. Values identified as outliers by the statistical tests performed in the Reference Value Advisor software were removed, and the other data from those reference individuals were evaluated to determine if the full dataset from that patient should be excluded [21]. As a trend toward decreased albumin and increased globulins associated with normal egg‐laying has been identified in egg‐laying chickens, close evaluation was given to non‐outlier albumin values in individuals with at least 1 outlier globulin fraction [17].

Descriptive statistics, including median, minimum, maximum, mean, and standard deviation, were calculated for each measurand individually. Distribution was classified using both statistical methods (Anderson–Darling test, statistical significance *p* < 0.05 for *n* > 65, *p* < 0.25 for *n* < 65) and by visual evaluation of histograms [22, 23]. When appropriate, the 95% RIs were calculated using nonparametric statistical methods, with 90% CI calculated using bootstrap methods as the preferred method. Due to disparities in sample size and dataset distribution, the Mann–Whitney test was used to compare results between hens and roosters. The statistical need for partitioning based on sex was evaluated using the method recommended by Lahti. Specifically, partitioning is needed if > 4.1% or < 0.9% of a subgroup is outside the combined RI, and partitioning could be appropriate if between 3.2%–4.1% or 0.9%–1.8% of a subgroup is outside the combined RI [24].

Active egg laying impacts the protein profile to an extent that can be detected in the electrophoretic profile. To account for this effect on RI data, electrophoretograms from hens that were consistent with egg laying were identified by a single observer (ARM), and RIs were calculated based on this partitioning. As the distinction between the partitioned groups was made post hoc based on electrophoretic profile, statistical evaluation between groups was not investigated.

Statistical analyses were conducted using Microsoft Excel (Microsoft Office 2016; Microsoft) and the Reference Value Advisor macro, GraphPad Prism 9 (GraphPad Software Inc) [21]. Alpha was set at 0.05, unless otherwise stated.

## 3 Results

The measured CV_%_ for all tested measurands in chickens was < 8% and was deemed acceptable (Table 2). The assays were determined to be performing sufficiently for a single measurement of each sample.

**TABLE 2:** Observed analytic variation in duplicate measurement of chicken plasma samples.

	Units	Chicken		
		Mean	SD	CV%
Total protein	g/dL	5.87	0.25	4.2
Alb_PE_	g/dL	1.722	0.038	2.2
Alpha‐1	g/dL	0.380	0.014	3.7
Alpha‐2	g/dL	0.810	0.006	0.7
Beta‐1	g/dL	0.832	0.012	1.4
Beta‐2	g/dL	1.036	0.046	4.4
Gamma	g/dL	0.558	0.043	7.7
A:G_PE_		0.476	0.010	2.1

**FIGURE 1:**
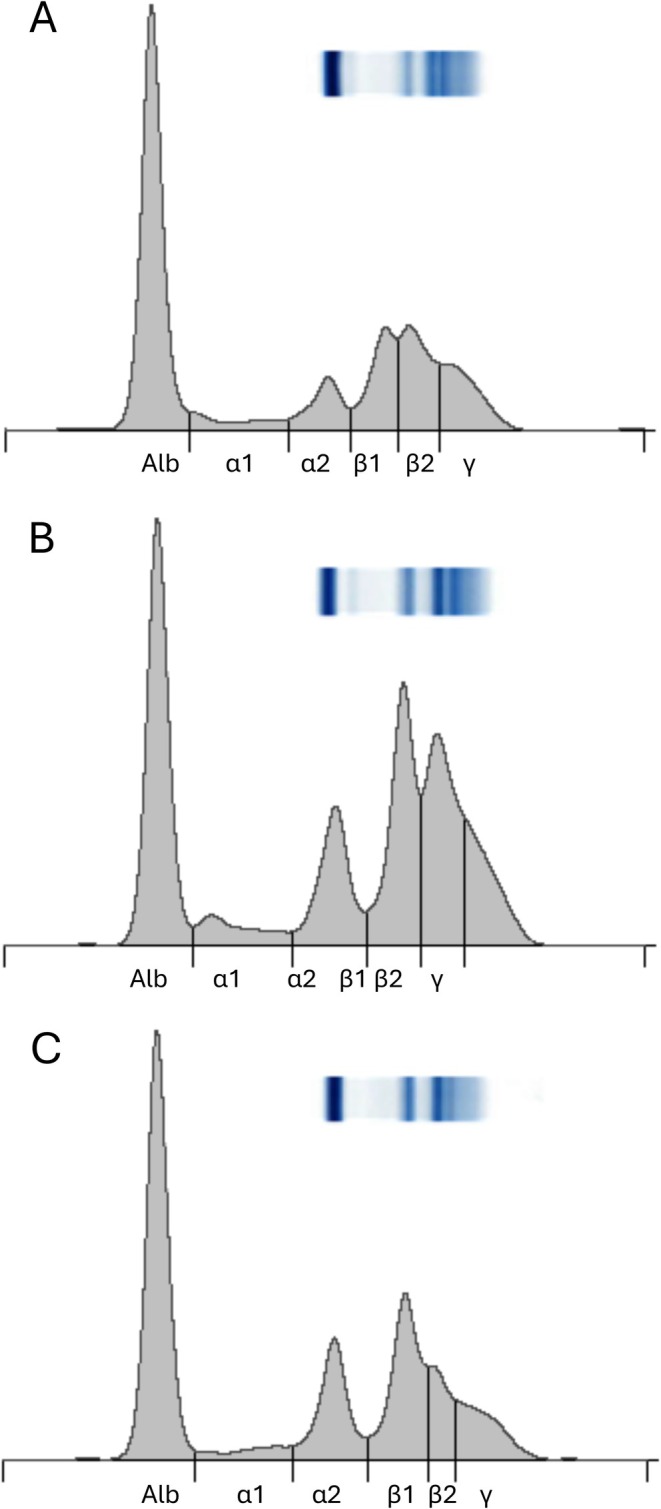
Chicken plasma protein electrophoretogram demonstrating morphology and placement of fraction demarcations. (A) Adult non‐molting hen, total protein = 5.3 g/dL. (B) Adult non‐molting hen with morphology suggestive of active egg‐laying, total protein = 6.7 g/dL. (C) Adult non‐molting rooster, total protein = 4.8 g/dL.

Agarose gel electrophoresis was completed for all samples. The electrophoretograms were divided into 6 fractions (Figure 1). Evaluation of the full dataset from patients with a single outlier did not demonstrate an electrophoretic pattern of subclinical disease nor suggest the need for removal of the full dataset from that patient. Only the values identified as outliers by statistical methods were excluded, and the remaining values from those patients were retained. Reference intervals were calculated for all chickens (Table 3).

**TABLE 3:** Reference intervals for plasma proteins in chickens measured using agarose gel electrophoresis.

Measurand	Units	Sex	*n*	Mean	SD	Median	Min	Max	Aderson–Darling *p*‐value	Distribution^a^	Method^b^	LRL of RI	URL of RI	CI 90% of LRL	CI 90% of URL	Mann–Whitney *p*‐value
Total protein	g/dL	All	117	5.2	0.7	5.2	3.4	7.1	0.38	G	NP	3.8	6.9	3.4–4.1	6.5–7.1	
		Hen	91	5.4	0.7	5.3	3.9	7.1	0.14	NG	NP	4.0	7.0	3.9–4.3	6.7–7.1	< 0.001
		Rooster	24	4.7	0.5	4.7	3.9	6.2	0.291	G	P	3.6	5.9	3.3–3.9	5.5–6.2	
Albumin	g/dL	All	117	2.16	0.36	2.19	1.25	3.05	0.873	G	NP	1.33	2.91	1.25–1.55	2.74–3.05	
		Hen	91	2.22	0.33	2.23	1.40	3.05	0.486	G	NP	1.47	2.92	1.40–1.62	2.75–3.05	< 0.001
		Rooster	23	2.04	0.33	1.91	1.68	2.81	0.007	NG	R	1.18	2.67	1.00–1.43	2.40–2.98	
Total alpha	g/dL	All	114	0.79	0.14	0.79	0.42	1.16	0.246	G	NP	0.52	1.09	0.42–0.59	1.02–1.16	
		Hen	94	0.79	0.16	0.77	0.42	1.24	0.02	NG	NP	0.52	1.20	0.42–0.58	1.05–1.24	0.077
		Rooster	20	0.81	0.06	0.81	0.7	0.92	0.623	G	P	0.68	0.93	0.65–0.72	0.89–0.97	
Alpha‐1	g/dL	All	120	0.24	0.09	0.22	0.09	0.51	0.000	NG	NP	0.13	0.47	0.09–0.14	0.40–0.51	
		Hen	96	0.25	0.09	0.23	0.09	0.51	0.000	NG	NP	0.13	0.47	0.09–0.14	0.40–0.51	0.007
		Rooster	21	0.18	0.04	0.18	0.14	0.27	0.08	NG	P	0.11	0.26	0.09–0.13	0.24–0.28	
Alpha‐2	g/dL	All	116	0.56	0.14	0.54	0.28	0.93	0.001	NG	NP	0.33	0.91	0.28–0.37	0.81–0.93	
		Hen	93	0.53	0.14	0.50	0.28	0.93	0.000	NG	NP	0.33	0.86	0.20–0.36	0.80–0.93	< 0.001
		Rooster	20	0.65	0.09	0.64	0.49	0.91	0.003	NG	R	0.44	0.82	0.37–0.54	0.74–0.90	
Total beta	g/dL	All	112	1.64	0.48	1.58	0.48	2.88	0.033	NG	NP	0.85	2.74	0.48–0.92	2.64–2.88	
		Hen	93	1.77	0.56	1.67	0.65	3.09	0.004	NG	NP	0.90	3.04	0.65–1.00	2.90–3.09	< 0.001
		Rooster	20	1.44	0.22	1.39	1.05	1.88	0.139	NG	R	0.91	1.88	0.83–1.08	1.72–2.06	
Beta‐1	g/dL	All	120	0.83	0.32	0.81	0.15	1.63	0.008	NG	NP	0.24	1.50	0.15–0.42	1.40–1.63	
		Hen	96	0.82	0.22	0.73	0.22	1.63	0.000	NG	NP	0.30	1.56	0.22–0.42	1.43–1.63	0.286
		Rooster	21	0.89	0.18	0.92	0.56	1.3	0.478	G	P	0.50	1.29	0.39–0.62	1.16–1.40	
Beta‐2	g/dL	All	116	0.87	0.37	0.82	0.26	1.86	0.002	NG	NP	0.33	1.79	0.26–0.38	1.56–1.86	
		Hen	96	1.00	0.40	0.93	0.37	1.99	0.001	NG	NP	0.39	1.97	0.37–0.48	1.81–1.99	< 0.001
		Rooster	22	0.48	0.12	0.48	0.26	0.75	0.958	G	NP	0.22	0.75	0.16–0.30	0.65–0.80	
Gamma	g/dL	All	110	0.50	0.15	0.47	0.21	0.83	0.016	NG	NP	0.26	0.82	0.21–0.27	0.78–0.83	
		Hen	91	0.54	0.17	0.51	0.26	1.03	0.003	NG	NP	0.26	1.00	0.26–0.30	0.82–1.03	0.001
		Rooster	21	0.38	0.10	0.38	0.21	0.62	0.867	G	P	0.17	0.60	0.11–0.23	0.53–0.67	
A:G		All	119	0.73	0.21	0.73	0.19	1.32	0.692	G	NP	0.24	1.20	0.19–0.42	1.09–1.32	
		Hen	96	0.73	0.23	0.35	0.19	1.32	0.778	G	NP	0.24	1.21	0.19–0.41	1.23–1.32	0.844
		Rooster	23	0.73	0.12	0.72	0.53	0.94	0.667	G	P	0.48	0.98	0.41–0.55	0.91–1.05	

^a^ Anderson–Darling: G, Gaussian; NG, non‐Gaussian.

^b^ P, parametric; NP, nonparametric; R, Robust.

Mann–Whitney test suggested statistical differences (*p* < 0.05) between hens and roosters for all measurands except total Alpha globulin, *p* = 0.08, beta‐2 globulin, *p* = 0.29, and A:G, *p* = 0.84 (Table 3). When the hen data were evaluated for partitioning from roosters using the method recommended by Lahti, none of the measurands had between 1.8% and 3.2% of the individuals outside the combined reference intervals. Based on this and the expected sex differences, the data were partitioned by sex, statistical and visual identification of outliers for exclusion was completed, and the individual protein values identified as outliers were excluded from the partitioned data. Partitioned RIs were calculated (Table 3).

Evaluation of the hen electrophoretograms identified 38/96 tracings that matched previously published data consistent with egg‐laying. The partitioned hen RI data is presented in Table 4.

**TABLE 4:** Reference intervals for plasma proteins in hens measured using agarose gel electrophoresis and after partitioning based on electrophoretic suggestion of egg‐laying.

Measurand	Units	Profile	*n*	Mean	SD	Median	Min	Max	Anderson–Darling *p*‐value	Distribution^a^	aMethod^b^	LRL of RI	URL of RI	CI 90% of LRL	CI 90% of URL
Total protein	g/dL	Non‐laying	58	5.2	0.6	5.2	3.9	6.5	0.908	G	NP	3.9	6.3	3.9–4.3	6.0–6.5
		Laying	36	5.8	0.9	5.7	4.1	7.7	0.223	NG	R	4.1	7.9	3.5–4.3	7.2–8.1
Albumin	g/dL	Non‐laying	54	2.32	0.23	2.31	1.84	2.81	0.733	G	NP	1.87	2.80	1.84–1.99	2.70–2.81
		Laying	36	1.92	0.40	1.99	0.83	2.6	0.214	NG	R	1.15	2.82	0.90–1.38	2.61–2.98
Total alpha	g/dL	Non‐laying	59	0.75	0.15	0.72	0.42	1.16	0.008	NG	NP	0.47	1.09	0.42–0.57	0.99–1.16
		Laying	33	0.83	0.13	0.83	0.59	1.09	0.806	G	P	0.56	1.10	0.50–0.63	1.04–1.17
Alpha‐1	g/dL	Non‐laying	58	0.23	0.07	0.20	0.09	0.40	0.003	NG	NP	0.11	0.40	0.09–0.14	0.36–0.40
		Laying	36	0.28	0.09	0.27	0.15	0.51	0.0252	G	P	0.10	0.45	0.06–0.14	0.42–0.50
Alpha‐2	g/dL	Non‐laying	51	0.47	0.08	0.46	0.28	0.70	0.349	G	NP	0.30	0.69	0.28–0.34	0.62–0.70
		Laying	35	0.58	0.15	0.55	0.36	0.93	0.180	NG	R	0.25	0.88	0.18–0.32	0.78–0.96
Total beta	g/dL	Non‐laying	59	1.49	0.36	1.49	0.65	2.23	0.930	G	NP	0.77	2.25	0.65–0.95	2.07–2.26
		Laying	36	2.36	0.56	2.27	1.50	3.44	0.074	NG	R	1.16	3.53	0.92–1.39	3.24–3.77
Beta‐1	g/dL	Non‐laying	56	0.62	0.18	0.59	0.22	1.03	0.142	NG	NP	0.23	1.03	0.22–0.39	0.92–1.03
		Laying	36	1.11	0.30	1.15	0.50	1.63	0.519	G	P	0.51	1.72	0.37–0.66	1.57–1.87
Beta‐2	g/dL	Non‐laying	60	0.85	0.29	0.86	0.37	1.49	0.356	G	NP	0.37	1.47	0.37–0.42	1.35–1.49
		Laying	36	1.24	0.44	1.20	0.55	1.99	0.035	NG	R	0.31	2.15	0.04–0.49	1.97–2.52
Gamma	g/dL	Non‐laying	57	0.48	0.12	0.46	0.26	0.77	0.062	NG	NP	0.26	0.75	0.26–0.29	0.70–0.77
		Laying	35	0.65	0.21	0.64	0.28	1.15	0.085	NG	R	0.20	1.05	0.09–0.29	0.92–1.18
A:G		Non‐laying	59	0.86	0.16	0.83	0.48	1.21	0.120	NG	NP	0.51	1.21	0.48–0.62	1.14–1.21
		Laying	35	0.52	0.12	0.52	0.24	0.73	0.364	G	P	0.27	0.77	0.22–0.33	0.71–0.83

^a^ Anderson–Darling: G, Gaussian; NG, non‐Gaussian.

^b^ P, parametric; NP, nonparametric; R, Robust.

## 4 Discussion

This study generated RIs for electrophoretic protein fractions in apparently healthy backyard chickens in northern Colorado. Our PPE RIs do not include individuals in molt and are likely not representative of healthy molting individuals; molting has been shown to induce increased pre‐albumin and alpha‐1 and decrease other fractions in other species [25]. The sampled flocks were of non‐molting adult birds of mixed breeds, sampled without knowledge of egg‐laying status, and housed at high altitudes [17]. While these parameters reflect the demographic of healthy adult birds submitted for evaluation, some of these factors may impact the evaluated measurands and may lead to wider RIs. For example, high altitude has been shown to affect the growth of commercial flocks; therefore, the utility of our PPE RIs for backyard flocks housed closer to sea level should be evaluated [26]. The clinical utility of the PPE RIs should be evaluated in a cohort of chickens to test the ability of the reference intervals to detect a wide variety of clinical conditions.

Total protein reference intervals in this cohort were similar to those found in a previous study from our laboratory (3.6–6.5 g/dL) and from a cohort of backyard chickens in Washington state, USA (3.9–7.0 g/dL) [8]. We observed higher protein fractions in hens than in roosters, likely due to egg‐laying.

The samples were frozen at −20°C between total protein measurement and electrophoresis. Freezer storage prior to electrophoretic evaluation is common in clinical and research settings when batch analysis is pursued. When statistically significant effects of freezer storage are reported, they are typically clinically negligible [27, 28, 29, 30]. Nonetheless, the RIs generated in this study may not perfectly reflect samples in which total protein and electrophoresis are performed without freezing.

As could be expected from the literature, the electrophoretic albumin RIs derived in this study were different from reported albumin RIs developed using dye‐binding methods, although no consistent pattern was observed [4, 5, 6, 7]. The electrophoretic albumin RI (1.33–2.91 g/dL) was lower than the dye‐binding albumin RIs observed in the cohort from Washington state (1.5–3.3 g/dL) and higher than the RI previously derived in our lab (0.9–2.1 g/dL) [3, 8]. As different analyzers and methods were used for these evaluations, meaningful comparisons are difficult; however, the data suggest that method‐specific albumin RIs are warranted. A method comparison of electrophoretic and dye‐binding albumin assessment was completed using these samples and is published elsewhere [31].

Previously published chicken protein electrophoretic RIs did not use current statistical approaches, and some were compiled using markedly different electrophoretic techniques than those used in this work, making meaningful comparison of the data challenging [12, 13, 14, 15]. The electrophoretic technique can significantly impact the electrophoretic location of proteins, including those in chickens [15]. Pre‐albumin was not noted as a distinct electrophoretic band in our samples, but pre‐albumin has been reported in other studies, including studies that reportedly used the same method as was used in this study [15, 16, 17, 32]. This may suggest an impact from differences in breeds, diet, housing, environment, or some other factor, and argues for the generation of lab‐specific RIs.

Given the considerable variation in electrophoretic patterns between species, differences between the chicken globulin fraction RIs with those published for other species are not unexpected [6, 7, 10, 11, 20]. However, the data suggest that species‐specific PPE RIs are warranted.

Current guidelines do not explicitly address the handling of the full dataset from patients with an outlier value identified in a single measurand. Generally, retaining suspected outliers is recommended when the health status of the reference individuals is known, and exclusion is wise when the health status is less well established, such as when sampling wildlife [18]. Exclusion of concurrent data biologically related to identified outlier values suggestive of subclinical illness is reasonable. For example, in an individual with an outlier for total white blood cell concentration, it may not be necessary to eliminate biochemistry data, but one might wisely remove the leukocyte differential counts from the dataset for that individual if one assumes that the total WBC outlier represents an abnormality in leukocytes. A relationship between some protein fractions can be expected, such as an acute phase response, inducing hypoalbuminemia concurrent with positive acute phase or chronic inflammatory changes, inducing increases in various globulin fractions. In this study, evaluation of the cases with at least 1 outlier, including close evaluation of albumin fractions, failed to suggest the need to exclude all data from any patient, and the non‐outlier data from these cases were retained. Similarly, guidance for handling the full dataset after statistically identified outliers are identified in the partitioned data is not included in the guidelines. Partitioning allows evaluation of the data distribution of a subpopulation without influences from a separate but numerically overlapping subpopulation. This allows identification of suspected outliers of one subpopulation that lie within the middle of the other subpopulation's distribution. Removal of these values near the central mass of the data would have a limited impact on the calculation of the upper and lower reference limits of the non‐partitioned data using nonparametric methods, a process that focuses on the data with the highest and lowest percentiles and essentially ignores data within the center of the distribution. For example, in this study, roosters had a lower beta‐2 fraction (median = 0.48 g/dL) than hens (median = 0.93 g/dL). Two roosters had beta‐2 values of 1.11 g/dL and 1.01 g/dL, identified as outliers only after evaluation of partitioned data, but these were close to the median beta‐2 value for hens and all individuals, 0.82 g/dL, and therefore were not relevant to the calculation of the non‐partitioned beta‐2 upper reference limit, 1.79 g/dL. Additionally, partitioning allows recognition that values identified as outliers when all data are considered may appropriately fit within the expected distribution of a subpopulation, may not be evidence of subclinical illness, and might appropriately be retained. For example, 4 hens had beta‐2 fraction values that were statistically identified as outliers when all values were considered, but these values were not identified as outliers when only hens were considered. Retention of the data from these 4 hens caused an increase in the beta‐2 upper reference limit after partitioning (1.97 g/dL). This study did not use the outlier status of partitioned data to exclude or retain values from the non‐partitioned dataset. The approach to handling statistically identified outliers in this study may have retained values impacted by subclinical illness, egg laying, or other factors. However, it is worth noting that the reference intervals for hens produced using this protocol are substantially similar to recently published electrophoretic protein values for hens, recognizing that a parametric method (mean ± 2*SD) to estimate the limits of the central 95% of the population of values reported by Tithova for laying hens [17] would be expected to roughly match a nonparametrically derived reference interval. This suggests that outlier handling had minimal effect on the resulting reference intervals.

Evaluation for partitioning suggested statistical differences between sexes for essentially all electrophoretic measurands. However, most of the differences were small and likely not of clinical significance, suggesting the use of non‐partitioned RI may be acceptable clinically. Notably, there were larger mean differences between the sexes for albumin, beta‐2, and gamma globulin. This likely reflects changes in protein metabolism associated with egg laying, as albumin, gamma globulin, and lipoprotein (often found in the beta region, depending on the electrophoretic technique used) are incorporated into eggs [32, 33]. As recently demonstrated, these fractions may be impacted by the stage of laying, and there is significant variation between individuals, even when a single period of egg‐laying is considered [17]. An attempt was made to partition the electrophoretic profiles based on evidence of egg‐laying and produce RI. This likely failed to accurately identify all egg‐laying hens, as fewer hens had evidence of egg‐laying when sampled during summer, when egg‐laying is expected. While the RIs partitioned this way are narrower, they likely do not capture the variation that can be seen with laying activity. This may impact the utility of the partitioned RIs; further evaluation of RI partition based on the known stage of laying is appropriate.

Direct comparison of electrophoretic pattern morphology and associated reference internals between studies is challenging due to expected differences caused by the choice of technique [34]. However, a pattern is evident in other galliform protein electrophoresis profiles, characterized by a predominance of albumin and higher proportions of beta globulins relative to alpha fractions, as observed in this study. For instance, similar patterns can be inferred from the data presented by Roman et al. for peafowl and Lynch and Stafseth for turkeys [35, 36]. To enable a more comprehensive comparison of protein electrophoresis profiles, further studies using a consistent electrophoretic technique for all samples, larger sample sizes, and individuals from multiple galliform species are necessary. Such research would provide a broader dataset to enhance the validity of cross‐species comparisons.

This study generated PPE RIs for backyard chickens in northern Colorado according to current guidelines. These RIs can be used to assist in health assessments and aid in the care of chickens kept as food‐producing pets.

## Conflicts of Interest

The authors declare no conflicts of interest.
